# Cancer care in Needle Hospital, Hargeisa, Somaliland

**DOI:** 10.3332/ecancer.2023.1642

**Published:** 2023-11-30

**Authors:** Gebrekirstos Hagos, Nazik Hammad, Susannah Stanway, Abdikani Yusuf, Tekleberhan Hailemariam

**Affiliations:** 1 Needle Hospital, Hargeisa 90203, Somaliland; 2 Saint Michael’s Hospital, University of Toronto, Toronto M5B 1W8, Canada; 3 Royal Marsden Hospital, Sutton Surrey SM2 5PT, UK; 4 Dire Dawa University, Dire Dawa 1362, Ethiopia

**Keywords:** cancer care, Needle Hospital, Somaliland

## Abstract

Somaliland is an autonomous region in the northern part of Somalia that declared its independence in 1991. It is a low-income country (LIC) with a population size of 5.7 million with a gross domestic product per capita of $775. Health services are delivered by public, private and non-governmental organisations. The public health care system in Somaliland is facing huge challenges. Seven percent of the population suffers from non-communicable diseases, but data on cancer incidence and mortality are not available. Much of the emphasis in public health has been placed on primary care and maternal and child health. There is still a large gap in cancer prevention, early detection and screening in the country. Additionally, there is no cancer registry or published data on cancer. Currently, there are a few private hospitals that provide chemotherapy services in Somaliland of which Needle Hospital is one. Services provided in this hospital include medical oncology for all solid tumours, palliative care, follow-up and cancer health education. The hospital provides services for patients from Somaliland and neighbouring countries including Djibouti, Somalia and Ethiopia. As a new oncology clinic in an LIC, the clinic is facing many challenges, like the absence of a multidisciplinary tumour board, presentation of patients at the advanced stage of tumours and poor cancer awareness in the general population.

## Background

Somaliland is an autonomous region in the northern part of Somalia that declared its independence in 1991. However, internationally, Somaliland is not recognised as an independent nation. It shares boundaries with the Gulf of Aden in the north, Somalia in the east, Ethiopia in the southwest and Djibouti in the northwest. It has six administrative regions. Its population size is 5.7 million, with the majority of the population living in urban centres [1, 2]. According to the Ministry of Health Development, life expectancy for males is 54 and that of females is 57 years [1]. Somaliland is a low-income country (LIC) with a gross domestic product per capita of $775 [3].

## Introduction

Globally, the incidence and mortality from cancer are rapidly increasing, and it is a major public health problem in many LICs [4, 5]. Worldwide, an estimated 19.3 million new cancer cases and almost 10.0 million cancer deaths occurred in 2020 [6]. Based on global cancer observatory (GLOBOCAN's) estimation, the global cancer burden is expected to be 28.4 million cases in 2040, a 47% rise from 2020 [7]. The aetiology behind the rising cancer burden in low- and middle-income countries (LMICs) is likely to be multifactorial and include changes in demographics and lifestyle. The higher proportion of patients with cancer in LMICs who have advanced-stage disease at the time of diagnosis has led to a greater case fatality rate in LMICs compared with high-income countries [8]. To date, cancer has been a low public health priority in Africa. This is due to limited health resources and other pressing public health issues such as malaria, tuberculosis and acquired immune deficiency syndrome [9, 10]. The purpose of this article is to provide an overview of current cancer care services in Somaliland and at Needle Hospital in particular and put forward recommendations to the Ministry of Health Development of the Republic of Somaliland.

### Health care in Somaliland

Somaliland’s health service delivery is structured around the Essential Package of Health Services framework. Health care services are delivered through five tiers: the community level, the primary health unit, the health centre, the referral health centre/district hospital and regional hospitals. The services are delivered by public, private and nongovernmental organisations (NGOs). The public health care system in Somaliland faces huge challenges. The main challenges are financial constraints, human resource capacity, limited infrastructure, donor dependency and a fragmented health system [11]. The government of Somaliland is driven to strengthen the health system despite facing multiple challenges in its efforts to improve coverage, access, staffing and service delivery. Much of the emphasis has been placed on primary care and maternal and child health [12]. Information on the state of non-communicable diseases (NCDs) in Somaliland is scarce, and little is known regarding the impact of NCDs and their evolution over time. Some information on NCDs is available from the 2020 report of the Somaliland Health and Demographic Survey. Based on this data, 7% of the population suffers from NCDs [13]. Cancer intelligence is vital to plan a country’s cancer services, yet precise data on cancer incidence and mortality is unavailable because a cancer-specific population-based registry is not available. Based on the GLOBOCAN 2020 report, there were 10,134 new cancer cases in Somalia, where breast, cervical and colorectal cancers were the leading types of cancer, accounting for 18.7%, 10.4%, and 7.3%, respectively [14]. But to the best of our knowledge, there has been no incidence or pattern of cancer reported in Somaliland. In addition, Somaliland has no national cancer control plan.

### Current status of cancer diagnosis and treatment in Somaliland

Cancer care requires a multidisciplinary team, ideally with a radiologist, surgeon, pathologist, medical oncologist, radiation oncologist, oncology nurse and social worker. Radiology is essential in providing quality cancer care [15]. In Somaliland, there are a few hospitals with cross sectional imaging (computed tomography or magnetic resonance imaging scans), and most are concentrated in the capital city, Hargeisa. There is no position emission tomography scanner or interventional radiology. The pathology service, which started in 2013 at Needle Hospital, is relatively new [16]. In the last few years, private hospitals providing pathology services have increased, and the biggest public referral hospital, Hargeisa Group Hospital, has established pathology services. Until now, pathology services have been limited to routine cytopathology and histopathology. Immunohistochemistry (IHC), flow cytometry and more advanced molecular pathology techniques are not yet available for clinical use.

For patients diagnosed with cancer, the only partially available treatment in the country was surgical therapy. As there are no surgical and gynaecological oncologists, cancer surgery is performed by general surgeons. For this reason, complex surgical procedures like thoracic and hepatobiliary cancers are not available in the country. In addition, data on oncology outcomes and standards of surgery are not available.

Radiotherapy is one of the main components of cancer treatment and requires substantial capital investment including for trained professionals in several disciplines. There is a significant shortage of radiotherapy machines in LMICs, and more than 50% of patients requiring radiotherapy in these settings do not have access to treatment [17]. Like many Sub-Saharan African countries, Somaliland has no radiotherapy services [18]. Cancer patients who need radiation therapy as part of their care must travel abroad.

Currently, there is no public cancer treatment centre in Somaliland, and there are private hospitals that provide chemotherapy. Needle Hospital is one of them, and it started the service in June 2022.

## Cancer care in Needle Hospital

Needle Hospital, a multispecialty hospital established in 2013 [16], is located in Hargeisa, Somaliland. The first pathology services in Somaliland commenced here. Services at Needle Hospital include pathology, internal medicine, head and neck surgery, radiology, paediatrics and child health, a laboratory and a pharmacy. Pathology services in Needle Hospital include fluid cytology, bone marrow aspiration, fine needle aspiration cytology, peripheral morphology and histopathology.

**Figure 1.**
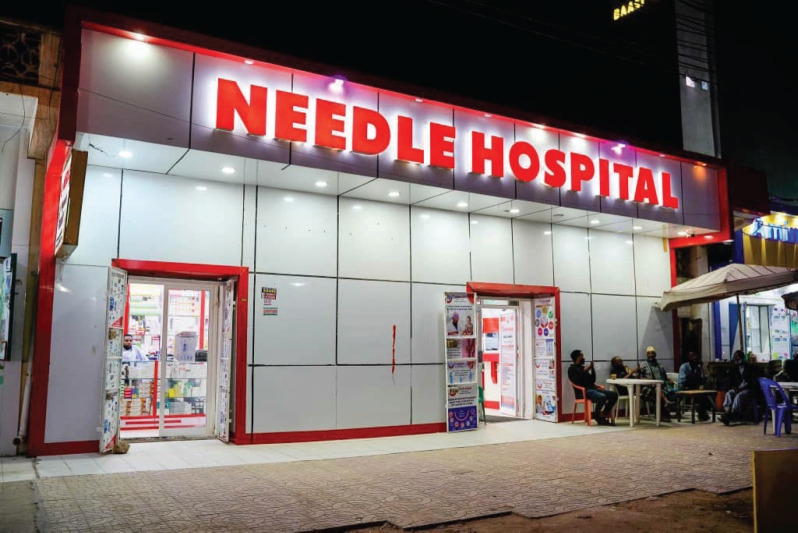
Needle Hospital Hargeisa, Somaliland (Main Branch).

**Figure 2.**
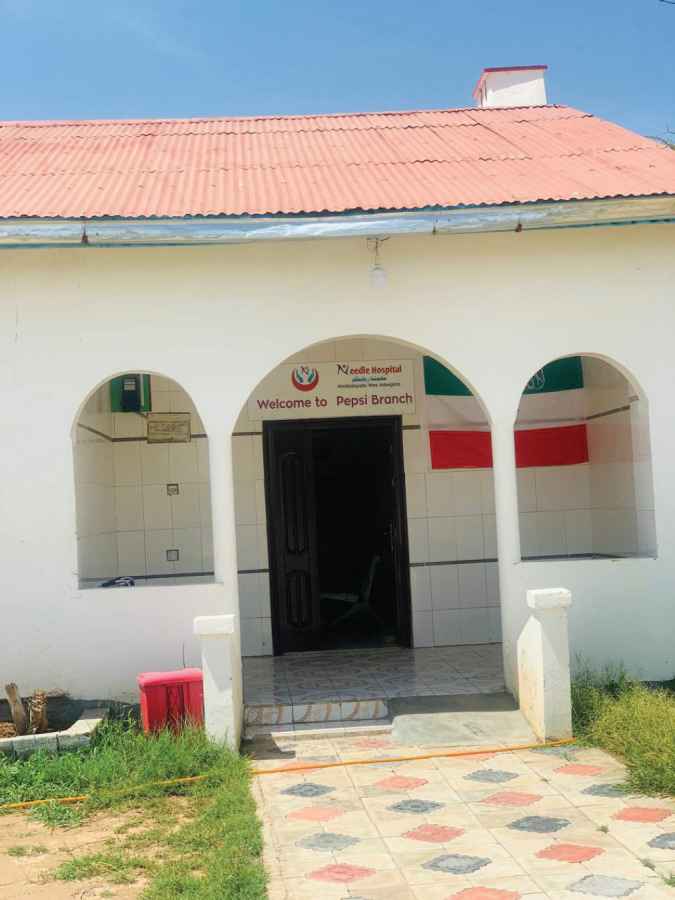
Needle Hospital, Hargeisa, Somaliland (Pepsi Branch).

Due to the strong commitment of the hospital management, a cancer treatment service at Needle Hospital, Hargeisa, was started in June 2022. This was initially in the main hospital, and on 1st October 2022, it moved to a separate facility in Hargeisa called, Needle Hospital Pepsi Branch (Figures 1 and 2). This centre is a dedicated cancer care clinic and has an outpatient clinic, male and female wards, along with a room dedicated to oncology emergencies. The clinic also has its own oncology pharmacy, laboratory and chemotherapy preparation room. The centre has six beds for chemotherapy administration, and it has one clinical oncologist and trained nurses. Services provided in the centre include medical oncology for all solid tumours, palliative care, follow-up and cancer health education. When radiotherapy is indicated, patients are referred abroad.

**Figure 3.**
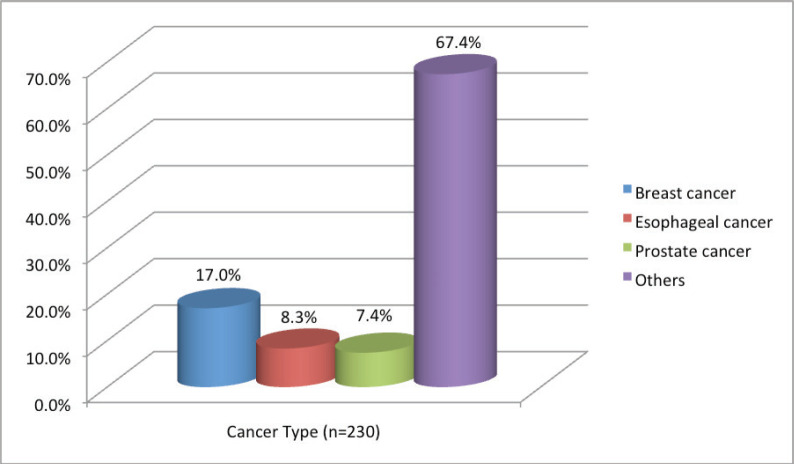
The three common solid cancers seen in Needle Hospital Cancer Clinic, Hargeisa, Somaliland from June 2022 to July 2023.

The clinic provides services for patients from Somaliland and neighbouring countries including Djibouti, Somalia and Ethiopia. The pharmacy has a supply of most chemotherapy agents used for the treatment of solid tumours, hormonal therapy for breast, prostate and thyroid cancers, and some targeted therapies. Immunotherapy is not yet available. To date, more than 230 patients of many cancer types have been evaluated and treated in the cancer clinic. The most common types of solid cancers seen in the clinic are breast, oesophageal and prostate cancers, accounting for 17.0%, 8.3% and 7.4%, respectively (Figure 3).

### Challenges of the cancer clinic

Being a new oncology clinic with limited resources, the clinic is facing many challenges, including:

- absence of a multidisciplinary tumour (MDT) board;
- advanced stage of the cancer at presentation;
- financial constraints on patients for investigation and management;
- poor cancer awareness among the general population;
- limitations to diagnostic capacity for example, lack of IHC; and
- constrained treatment options for example no immunotherapy, limited targeted therapies and absence of radiotherapy services.

## Discussion

Great strides have been made in advancing cancer care in Somaliland. A designated clinic has been established run by a specialist and patient data are being collected. Introducing a MDT board and ensuring continuous professional development of its members is a priority. Integrating palliative care within oncology services is imperative. Improving cancer literacy at the community level particularly on the areas of cancer prevention and early presentation will be very important and necessary. Future directions in collaboration with the Ministry of Health Development include strengthening the hospital-based cancer registry and establishing a more formal registry throughout the Republic that collects more sophisticated intelligence. Health system strengthening across the continuum which includes screening for common cancers, improving diagnostics such as starting IHC within pathology services, and establishing radiotherapy service are necessary. Collaborations are being built with regional and international colleagues, alliances, organisations including academic institutions, NGOs, United Nation agencies and beyond to work on these future directions in order to provide improved cancer services locally for all citizens of Somaliland.

## Conclusion

Despite facing significant challenges, Needle Hospital in Hargeisa, Somaliland, has made commendable strides in providing cancer care services to the community. The establishment of a dedicated cancer clinic and the provision of chemotherapy and palliative care represent important steps towards addressing the burden of cancer in the country. Addressing the challenges will require concerted efforts from the hospital, the Ministry of Health Development, and the international community. Solving these challenges and implementing the recommendations can improve cancer outcomes and reduce the burden on the population.

## List of abbreviations

GDP, gross domestic product; GLOBOCAN, global cancer observatory; IHC, immunohistochemistry; LIC, low-income country; LMIC, low-and middle-income countries; MDT, multidisciplinary tumour; NCD, non-communicable diseases; NGO, Non-Governmental Organisation.

## Conflicts of interest

The authors declare that they have no conflict of interest.

## Funding

No funding was received for this study.
